# The effect of psychological first-aid virtual education on the communication skills of nurses in disasters: a randomized controlled trial

**DOI:** 10.1186/s40359-024-01682-0

**Published:** 2024-04-08

**Authors:** Sayed Mohammad Sadegh Madani, Ali Bahramnejad, Zahra Farsi, Azizeh Alizadeh, Maryam Azizi

**Affiliations:** 1 Health in Disaster and Emergencies Department, Aja University of Medical Sciences, Tehran, IR Iran; 2 Neuroscience Research Center, Institute of Neuropharmacology, Aja University of Medical Sciences, Tehran, Iran; 3 Medical-Surgical Nursing, Research and Ph.D. Nursing Departments, Nursing School, Aja University of Medical Sciences, Tehran, Iran; 4 NEZAJA Health Department, Assistance of Mental Health in Khanevadeh Hospital, Tehran, IR Iran; 5 Health in Disaster and Emergencies Department, Faculty of Nursing, Aja University of Medical Sciences, Shareati Street, Tehran, IR Iran

**Keywords:** Virtual education, Psychological first-aid, Communication skills, Nurse, Disaster

## Abstract

**Background:**

Disasters have affected the physical and mental health of people around the world. Since nurses are frontlines in disasters, it seems necessary to prepare for this responsibility. This study investigates the effect of psychological first-aid virtual education on the communication skills of nurses in disasters such as COVID pandemic.

**Methods:**

In a randomized controlled trial, 55 nurses were selected by purposive sampling method from two hospitals in Isfahan and Tehran, Iran in December—November 2022 and randomly replaced in the intervention group who participated in psychological first aid virtual training and control group. The data were collected through the personal information form and Communication Skills –Test-Revised (CSTR).

**Results:**

Two groups were homogeneous in terms of communication skills (*p* = 0.177), the total score of communication skills was significant between the two groups after the intervention (*p* < 0.0001). Regarding communication skills, in the pre-intervention phase, the subscale of “the ability to receive and send messages” and “insight into the communication process” the difference before the intervention was not significant between the two groups (*p* > 0.05). However, it was significant between the two groups after the intervention (*p* < 0.05), and regarding “emotional control”, “listening skills”, and “communication along with assertiveness” the difference before and after the intervention was not significant between the two groups (*p* > 0.05).

**Conclusion:**

Pre-disaster training and virtual education can increase nurses’ communication skills in their ability to handle a disaster such as COVID pandemic. Virtual education of post-disaster psychological interventions is suggested.

**Trial registration:**

IRCT20220923056023N1; date: 2023–01-31.

## Background

Disasters are unpredictable, catastrophic, and increasing phenomena which significantly leave a large-scale of physical and psychological trauma or deaths, disasters also have potential impacts on putting a tremendous burden on hospitals and medical centers through the provision of urgent medical care [1–3]. Nurses as the first healthcare responders have specific roles in different aspects of the disaster management cycle, like identifying and mitigating risks, participating in preparedness programs, effective and timely response on the disaster scene, and implementing rehabilitation programs [2, 4].

Nursing is the art of providing care to patients through combining the knowledge of clinical activity and personal skills like interpersonal communication [5–7]. Providing information and feedback, giving hope to desperate patients, expressing support, lessening the patient's family tension, and interaction with other healthcare providers are all done through communication skills [8]. Effective communication is a crucial core component of effective nursing care, which increase the patients and their families satisfaction, as well as lessoning the workplace violence, and the pain, stress and anxiety of the patient during hospitalization [9–11].

Despite the effect of communication skills on the quality of nursing care, the results of various studies show that nurses have not been successful in communicating with patients and their families. Jolly et al. showed that nurses scored the lowest in communication skills from the patients' point of view. Also, based on Farahani’s findings, only 23% of the patients were satisfied with nurses’ communication skills [12–15]. Therefore, healthcare providers, including nurses, need to learn communication skills [16].

To establish professional and effective communication skills, nurses should understand patients’ psychological conditions to make them feel less psychological distress and improve their resilience capacity, namely, one of the major goals of psychological first-aid (PFA) [17, 18].

PFA is an effective, supportive, and practical guide for rescuers to support individuals exposed to severe stress during or immediately after a disaster [17]. Individuals without specialized mental health training, such as public health professionals and first responders, can learn address the needs [19]. Identifying the urgent needs of affected individuals, providing disaster situation information, connecting with a social support network, and helping them to be resilient, account for the vital roles of PFA in disasters, show the importance of PFA training [17, 20]. Although PFA was developed as a specific crisis-oriented mental health disaster intervention for assessing and alleviating acute distress, few studies have evaluated the efficacy and applicability of PFA in the field [21].

Acute respiratory syndrome of the corona virus 2 (SARS-CoV-2), was one of the influential disasters after the Second World War, which spread rapidly, caused a pandemic and affected all aspects of people's lives [22]. The emergence of COVID-19 has plunged the world into an unprecedented public health crisis. Iran like other countries has used response protocols to control the spread of the virus. Quarantine conditions lead to the closure of some public and education centers. Closure of educational institutions changed the traditional face-to-face teaching to the online.

Virtual education / learning (e.g., e-learning) is a scheme in which teaching and learning typically occur in separate environments where educators present course content via Learning Management System (LMS), multimodality, the Internet, and video conferencing [17]. The use of virtual learning courses, due to the flexibility of this educational system, increases the quality of learning, facilitates access to a large amount of information, and reduces educational costs; this way, it provides a good opportunity to expand the academic content and increase the depth of learning [23].

In nursing education, due to intensive work schedule, constrained time and need of free time to participate in training courses, it is very important to design and implement intensive courses with the least cost and maximum effectiveness [24, 25].

It has been known that disasters affect different parts of individuals' physical and mental health [26]; physical support is a priority for survivor care management. Psychological support, however, is often ignored and unprofessional support provided. Due to the increase in earthquakes, floods, fires, and infectious diseases, the need for psychological and emotional care seems indispensable [27, 28].

It is recognized that patients and their caregivers positively value professionals who attentively listen, delivered information, and communicate in an empathetic way [29, 30]. Accumulating evidence from trials have shown that nurses lack effective and sufficient communication skills due to inadequate training or inability to understand the importance of patient-centered communication.

The authors' investigations showed that the conducted studies have not directly investigated the impact of PFA on communication skills, and most of the studies conducted in this field have focused on self-efficacy, competency, and knowledge of psychosocial support principles. Similarly, recent studies in Iran showed that training PFA is rarely provided in disasters [31–33].

In view of these, nurses, as one of the main members of healthcare system, are at the forefront of supporting injured people [34]. Therefore, considering the implementation of virtual PFA training, and considering that post-disaster emotions are similar in communities, the priority of emotional needs and how they are met are different, in the present study, these questions will be answered whether virtual education in PFA can have a significant effect on improving the communication skills of nurses or not.

## Methods

The study adheres to CONSORT guidelines.

### Materials and methods

#### Study design

This randomized controlled trial with pre-post intervention design has been approved in Iranian Registry of Clinical Trials (ID: IRCT20220923056023N1; date: 2023–01-31).

#### Sample size and setting

The sample size, 23 nurses per group, was estimated based on the study of Laktarash et al. [35] at a 95% confidence level (α = 0.05) and an 80% test power. However, 26 nurses were recruited for each group to account for the possibility of a 10% dropout rate.

First, 94 nurses were recruited by the convenience sampling method. Then, 39 were excluded from the study because they did not meet the inclusion criteria (*n* = 30) and declined to participate (*n* = 9). Fifty-five of them were randomly divided into the intervention group (*n* = 28) and the control group (*n* = 27) (one side of the coin for the control group and the other side for the intervention group). Totally, nurses who worked in two hospitals in Tehran and Isfahan, Iran in December to November 2022 were recruited in this study and participated to intervention (*n* = 26), and control groups (*n* = 24). There was about 9% attrition in the study.

$$n= \frac{{\left({{\text{Z}}}_{1-\frac{\alpha }{2}}+{{\text{Z}}}_{1-\beta }\right)}^{2}(\delta {1}^{2}+\delta {2}^{2})}{{\left({\upmu }_{1}-{\upmu }_{2}\right)}^{2}}=\frac{{\left(1.96+0.84\right)}^{2}\left({\left(7.06\right)}^{2}+{\left(8.97\right)}^{2}\right)}{{\left(66.13-59.5\right)}^{2}}=23.24=23$$

**Fig. 1:**
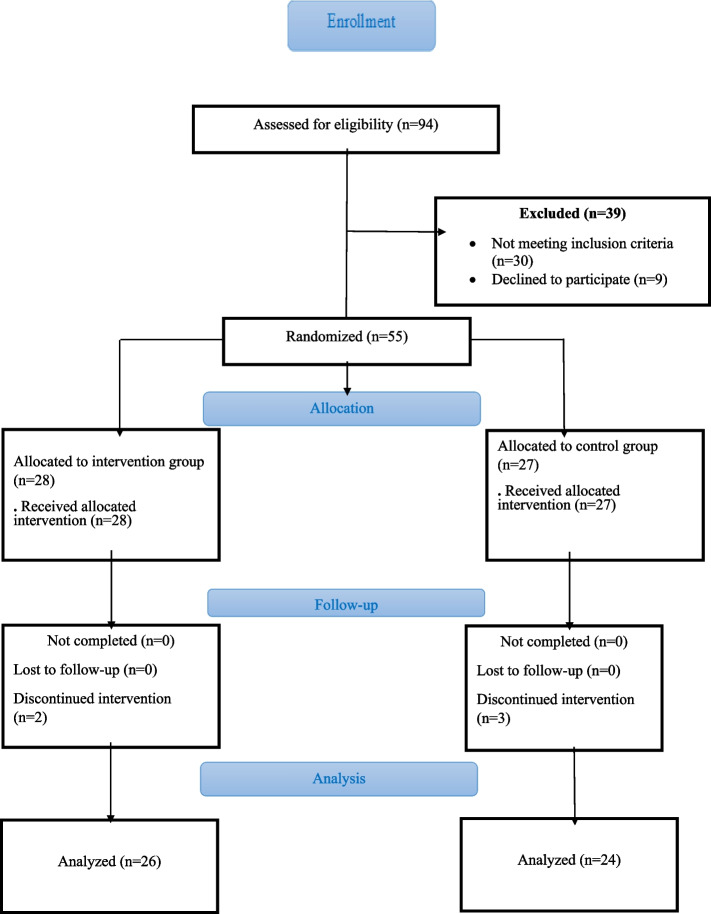
The study process

Inclusion criteria were: a bachelor’s or higher degree in nursing, not participating in crisis preparation courses before the study, having electronic communication devices (smartphones, tablets, laptops, etc.) to participate in virtual workshops, and also working as a nurse in one of the departments of hospitals in Tehran and Isfahan. Exclusion criteria were: not participating in more than one session of the virtual education workshop, incomplete answers to the questionnaires, being employed in the psychiatric departments of the sampled hospitals, and unwillingness to continue cooperation. Details are pigeonholed (Fig. 1).

#### Measurement

The two questionnaires of individual characteristics (age, gender, academic degree, and experience in disasters), and the Communication Skills Test—Revised (CSTR) were employed to gather data. The CSTR (2004) has 34 items. The person specifies the degree of conformity of his/her status with its content on a five-point Likert scale from never (1) to always (5). This questionnaire has five dimensions of “the ability to receive and send messages” (4, 5, 6, 12, 21, 22, 23, 24, 29), “emotional control” (7, 8, 9, 11, 13, 16, 28, 30, 32), “listening skills” (3, 25, 26, 27, 31, 34), “insight into the communication process” (1, 2, 17, 18, 20), and “communication along with assertiveness” (10, 14, 15, 19, 33). Each respondent receives a score between 34 and 170. Higher scores indicate better communication skills. Cronbach's alpha CSTR is equal to 0.69 and indicates acceptable internal consistency of this tool. Besides, the reliability coefficient of the instrument was obtained 0.71. Hosseinchari and Fadakar has confirmed the reliability of the items of this questionnaire with Cronbach's alpha method in a sample of 733 school students and university studentsas 0.71 and 0.69, respectively [36], and Yousefi (2006) found the reliability as 0.81 and validity as 0.77 using split half [37]. In this study, the Cronbach's alpha was 0.68.

#### Intervention and data collection

First, the educational content of PFA was collected based on the principles of PFA and the guidelines issued by the Ministry of Health, Treatment and Medical Education in Iran. The professors of the department of psychiatry and psychology of Isfahan and Kerman University of Medical Sciences confirmed the validity of the course. All training was conducted by the researchers with PFA training. In the intervention group, before the intervention, a face-to-face session was held to express the goals and introduce the nurses. Then, four one-hour virtual workshops were held for the intervention group. The instructor in the workshop were corresponding authors of the research who had a history of conducting psychological care seminars in disasters. Virtual meetings were held on the Sky room platform (Made in Iran, produced in 2017, this is a web hosting service, i.e. webinar, live online class and virtual seminar) for four weeks (one session per week). It was possible for nurses to ask questions about training (through virtual education, telephone, and social media). To encourage nurse engagement, internet fees for attending virtual training sessions were considered. After the training session, the questionnaires were virtually completed by nurses in the intervention group. However, the control group received training packages by the researchers for acknowledgment their participation in the research.

#### Content of training sessions

The content materials were used for training, under the permission of the professors of the department of psychiatry and psychology of Isfahan and Kerman University of Medical Sciences, based on the PFA principles and directives issued by the Iranian Ministry of Health and Medical Education.

The content materials included:

- The first session: Introduction, general explanation about the subject, and necessity of training (face-to-face education).
- The second session: Definition and classification of disasters and psychological consequences of disasters on affected people (virtual education).
- The third session: The basics of PFA, psychological reactions in different stages of disasters, and stress management (virtual education).
- The fourth session: Communication skills, preparing and getting information about the situation, and practical principles of PFA (virtual education).
- The fifth session: Providing appropriate support to victims, self-care strategies, and emergency mental health interventions (virtual education).

Besides, to prevent the transfer of content materials, the control group was examined first, and the intervention was implemented on the intervention group, and the interval between the pre& post- intervention was the same in both the intervention and control groups (four weeks). To comply with ethics and the possibility of access of other samples to education, files containing virtual educational content were provided to the control group.

#### Statistical analyses

Data were statistically analyzed using SPSS. Kolmogorov–Smirnov Test was employed to check the normality of the data in each group. The Chi-square, Fisher's exact test, and independent *t*-test were used to check the homogeneity of the two groups in terms of individual characteristics. Paired *t*-tests and Wilcoxon test were used to compare the mean score of nurses' communication skills before and after the intervention in each group. Besides, independent *t*-test and Mann–Whitney *U* was used to compare the mean score of nurses' communication skills in both groups. *P* < 0.05 was considered as a significant level.

#### Ethical considerations

The Ethics Committee of Aja University of Medical Sciences, Tehran, Iran approved this study (No. IR.AJAUMS.REC.1401.092). All principles of the Declaration of Helsinki were followed. Written informed consent was obtained from the nurses. Participation in the study was voluntary and the nurses could withdraw from the study at any time, and they were also assured about the confidentiality of the information related to the sample members.

## Results

The mean and standard deviation of the age of the nurses and the work experience of the nurses were 28.58 ± 4.57 (23 to 44 years) and 5.66 ± 5.25 (1 to 21 years), respectively. The majority of nurses were female (66%). Besides, the majority of participants (82%) had a bachelor’s degree in nursing. Both the intervention and control groups were homogeneous in terms of individual characteristics (age, gender, degree and experience in disasters) (Table 1).

**Table 1:** Comparison of individual characteristics of nurses in the Intervention and control groups

Variable	Intervention group (*n* = 26)	Control group (*n* = 24)	Test Statistics, df, *p*-value
	**Mean (SD)**	**Mean (SD)**	
**Age**	29.23 (5.09)	27.88 (3.94)	Independent sample *t*-test
			*t* = 1.048
			df = 48
			*p* = 0.300
	**f (%)**	**f (%)**	
**Gender**			Fisher's exact test
Male	9 (34.6)	8 (33.3)	value = 0.009
Female	17 (65.4)	16 (66.7)	*p* = 1.000
**Degree**			Fisher's exact test
BSc	24 (92.3)	17 (70.8)	X^2^ = 3.899
MSc ≤	2 (7.7)	7 (29.2)	df = 1
			*p* = 0.069
**Experience in disasters**			Fisher's exact test
Yes	6 (23.1)	2 (8.3)	X^2^ = 2.018
No	20 (76.9)	22 (91.7)	df = 1
			*p* = 0.250

*f* frequency, *SD* Standard Deviation

The independent *t*-test showed that the two groups did not have a significant difference in terms of the mean of “communication skills” before the intervention (*p* = 0.177), while this difference was significant after the intervention (*p* < 0.001). Besides, the paired *t*-test showed that the mean score of “communication skills” in the intervention group was not significantly different before and after the intervention (*p* = 0.057) and in the control group also, this score was not significantly different (*p* = 0.149) (Table 2).

**Table 2:** Comparison of the mean scores of communication skills dimensions of nurses in the intervention and control groups

**Variable**		**Intervention group** **(*n*** **= 26)**	**Control group** **(*n*** **= 24)**	**Independent sample ** ***t*** **-test** **Statistics, df, ** ***p*** **-value**
		**Mean)SD**	**Mean)SD**	
**Ability to receive and send massages**	Pre- intervention	31.69 (3.83)	30.08 (2.36)	*t* = 1.802 df = 42 *p* = 0.072
	Post- intervention	32.73 (3.46)	29.17 (3.10)	*t* = 3.824 df = 48 *p < 0.0001
	Paired *t*-test Statistics, df, *p*-value	*t* = -1.364 df = 25 *p* = 0.185	*t* = 1.357 df = 23 *p* = 0.188	
**Emotional control**	Pre- intervention	29.08 (3.03)	28.96 (3.14)	*t* = 0.136 df = 48 *p* = 0.893
	Post- intervention	29.73 (4.37)	27.58 (2.84)	(Mann–Whitney *U*) *u* = 231.5 *p* = 0.115
	Wilcoxon signed-rank test, *p*-value	z = -1.116 *p* = 0.280	z = -2.162 *p* = 0.029*	
**Listening skill**	Pre- intervention	20.23 (2.89)	19.79 (2.21)	*t* = 0.600 df = 48 *p* = 0.551
	Post- intervention	20.84 (2.27)	19.75 (2.13)	*t* = 1.754 df = 48 *p* = 0.086
	Paired *t*-test Statistics, df, *p*-value	*t* = -1.048 df = 25 *p* = 0.305	*t* = 0.085 df = 23 *p* = 0.933	
**Insight into the communication process**	Pre- intervention	17.65 (2.53)	16.58 (1.82)	*t* = 1.707 df = 48 *p* = 0.094
	Post- intervention	19.307 (1.67)	16.33 (1.63)	*t* = 6.364 df = 48 *p < 0.0001
	Paired *t*-test Statistics, df, *p*-value	*t* = -3.109 df = 25 *p = 0.005	*t* = 0.796 df = 23 *p* = 0.434	
**Communication along with assertiveness**	Pre- intervention	14.77 (2.49)	14.96 (1.76)	*t* = -0.308 df = 48 *p* = 0.759
	Post- intervention	14.46 (3.14)	14.67 (1.68)	*t* = -0.291 df = 39 *p* = 0.773
	Paired *t*-test Statistics, df, *p*-value	*t* = 0.527 df = 25 *p* = 0.603	*t* = 0.743 df = 23 *p* = 0.465	
**Total score of communication skills**	Pre- intervention	113.42 (8.62)	110.38 (6.92)	*t* = 1.371 df = 48 *p* = 0.177
	Post- intervention	117.08 (8.65)	107.50 (7.70)	*t* = 4.121 df = 48 *p < 0.0001
	Paired *t*-test Statistics, df, *p*-value	*t* = -1.995 df = 25 *p* = 0.057	*t* = 1.495 df = 23 *p* = 0.149	

*SD* Standard Deviation

*p* < 0.05*

The independent *t*-test revealed that the two groups did not have a significant difference in terms of the mean score “ability to receive and send message” before the intervention (*p* = 0.072), while this difference was significant after the intervention (*p* < 0.0001) (Table 2). Besides, the paired *t*-test showed that the mean score “ability to receive and send message” in the intervention group was not significantly different before and after the intervention (*p* = 0.185) and in the control group, this score was not significantly different (*p* = 0.188).

The independent *t*-test showed that the two groups did not have a significant difference in terms of the mean score of “emotional control” before the intervention (*p* = 0.893). Likewise, the mann–whitney *U*-test showed that this difference was not significant after the intervention (*p* = 0.115) (Table 2). The Wilcoxon test showed that the mean score of “emotional control” in the intervention group was not significantly different before and after the intervention (*p* = 0.280), but in the control group, this score was significantly different (*p* = 0.029).

The independent *t*-test revealed that the two groups did not have a significant difference in terms of the mean score of “listening skill” before the intervention (*p* = 0.551), also this difference was not significant after the intervention (*p* = 0.086) (Table 2). The paired *t*-test showed that the mean score of “listening skill” in the intervention group was not significantly different before and after the intervention (*p* = 0.305), and in the control group, this score was not significantly different (*p* = 0.933).

The independent *t*-test showed that the two groups did not have a significant difference in terms of the mean score of “insight into the communication process” before the intervention (*p* = 0.094), while this difference was significant after the intervention (*p* < 0.001). The paired *t*-test showed that the mean score of “insight into the communication prices” in the intervention group was significantly different before and after the intervention (*p* = 0.005), but in the control group, this score was not significantly different (*p* = 0.434) (Table 2).

The independent *t*-test showed that the two groups did not have a significant difference in terms of the mean score of “communication along with assertiveness” before the intervention (*p* = 0.759), and this difference was not significant after the intervention (*p* = 0.773). The paired *t*-test showed that the mean score of “communication along with assertiveness” in the intervention group was not significantly different before and after the intervention (*p* = 0.603) and in the control group, this score was not significantly different (*p* = 0.465) (Table 2).

## Discussion

In this study, the effect of virtual education on the communication skills of nurses in providing PFA in disasters such as COVID pandemic was investigated. The two intervention and control groups were homogeneous in terms of individual characteristics before the intervention. Two intervention and control groups, before and after the PFA virtual education, were assessed regarding the communication skills of nurses in the crisis. The results indicated that the communication skills score of the intervention group after receiving training were different between the two groups. In a study to investigate the relationship between communication skills, interpersonal ability and clinical competence of nursing students and to identify factors affecting clinical competence on 172 nursing students, factors affecting the clinical competence of nursing students included communication skills, interpersonal relationships, mental health status, and satisfaction with the nursing field [38]. In the present study, the sub-categories of communication skills, before the intervention, the average score between the two groups was homogeneous and no significant difference was found between the two groups. According to the results of the comparison between the two groups after the intervention, virtual training has had a positive effect on the two dimensions ("ability to receive and send message" and "insight into the communication prices”). In this regard, a study showed the effectiveness of education based on the BASNEF (Beliefs, Attitudes, Subjective Norms and Enabling Factors) model on the communication skills of nurses [39].

According to the results of the comparison between the two groups after the intervention, virtual training did not affect the three dimensions (“emotional control”, “listening skill”, and “communication along with assertiveness”). In support of the findings of this study, another study investigated that social competence training based on the Felner model has significantly improved the mean scores of communication skills after the intervention [35]. In the mentioned study in all sub-components of communication skills, the average scores increased after the intervention, but this average increase was significant only in the sub-component of listening skill at the 0.01 level. Also, another study showed that the implementation of the teaching method based on problem solving was effective on the verbal and non-verbal communication skills of nursing students [40]. In this study, all scores were checked immediately after the training sessions. Maybe if an opportunity was given to practice and the scores were checked again after some time, positive effects would appear for other dimensions.

The evidence have shown that the development and application of educational programs is important for increasing communication skills and interpersonal relationships and improves the clinical competence of nursing students [38]. A nurse's ability to communicate effectively is essential to build therapeutic relationships with patients and achieving greater patient satisfaction. It also minimizes medical errors and improves the quality of nursing care. Hospitals and healthcare systems must use agile methods to communicate information in an open and timely manner during periods of crisis [41]. Communication, especially in times of crisis, must convey clear and consistent messages [42]. In one study, five types of common professional skills were identified that nurses need to retrain, including casualty triage, observation and supervision, basic first aid techniques, psychological care, and communication skills [31]. Effective communication with patients, caregivers, and other medical staff is essential in effective nursing. Teaching communication skills for both student nurses and working nurses is one of the important goals of professional development and training in the nursing profession [22]. The spread of COVID-19 in the whole world has led the educational method from face-to-face to virtual education, which has its advantages and disadvantages [43]. Some of the advantages of virtual education includes reduction of economic costs in long term, flexibility and a suitable platform for teaching and learning, to promote health, prevent disease spread, improve self-management, and provide medical counseling and some of its disadvantages includes lack of sufficient skills in using virtual education, insufficient space, facilities and infrastructure, lack of effective interaction, lack of ethics, information security and the impossibility of quality evaluation, lack of proper software support, and the impossibility of proper attendance and absence [44, 45]. In another study, the advantages of using virtual education are its affordability, countering the rising cost of graduate education, the ability to provide world-class education to anyone with the ability to connect to a broadband connection [46]. However, due to the existence of COVID pandemic and the need to preserve the health of nurses, virtual education method was used in this study. It has also been tried to minimize the disadvantages of using this method by considering the appropriate application with easy use to provide training and create an interactive environment for the participants.

### Limitations

This study has limitations. For example, nurses had trouble accessing training materials due to slow internet speeds. This was, of course, solved by the efforts of the researchers and by holding the conference at a time when internet speeds were the fastest. Also, nurse access limitations due to busy work, lack of time, and fatigue during the COVID pandemic were addressed by considering the short duration of the intervention and tracking the impact of PFA training.

## Conclusion

Communication skills is one of the most important skills that nursing staff should receive to improve care in health systems. Therefore, it is necessary to give special importance to this part of education, which is given less attention. Considering the effectiveness of the PFA training of nursing and relief personnel that we achieved in this project, it is suggested policymakers to include PFA training in the curriculum of nursing students, especially students specializing in disasters, Also, its recommended to investigate the effects of PFA on psychosocial outcomes in affected individuals. It is suggested that due to the expansion of artificial intelligence and its ease of use, this method should also be used to improve the training of health workers.

## Data Availability

The datasets used for the current study are available from the corresponding author upon request.

## Abbreviations

COVID-19: Coronavirus disease 2019
PFA: Psychological first aid
CSTR: Communication skill test-revised
